# The Combination of DGT Technique and Traditional Chemical Methods for Evaluation of Cadmium Bioavailability in Contaminated Soils with Organic Amendment

**DOI:** 10.3390/ijerph13060595

**Published:** 2016-06-15

**Authors:** Yu Yao, Qin Sun, Chao Wang, Pei-Fang Wang, Ling-Zhan Miao, Shi-Ming Ding

**Affiliations:** 1 Key Laboratory of Integrated Regulation and Resource Development on Shallow Lakes, Ministry of Education, College of Environment, Hohai University, Nanjing 210098, China; yu2011358@163.com (Y.Y.); cwang@hhu.edu.cn (C.W.); mlz1988@126.com (L.-Z.M.); 2 State Key Laboratory of Lake Science and Environment, Nanjing Institute of Geography and Limnology, Chinese Academy of Sciences, Nanjing 210008, China; smding@niglas.ac.cn

**Keywords:** organic amendment, colza cake, cadmium bioavailability, DGT extraction, extraction method, plant

## Abstract

Organic amendments have been proposed as a means of remediation for Cd-contaminated soils. However, understanding the inhibitory effects of organic materials on metal immobilization requires further research. In this study colza cake, a typical organic amendment material, was investigated in order to elucidate the ability of this material to reduce toxicity of Cd-contaminated soil. Available concentrations of Cd in soils were measured using an *in situ* diffusive gradients in thin films (DGT) technique in combination with traditional chemical methods, such as HOAc (aqua regia), EDTA (ethylene diamine tetraacetic acid), NaOAc (sodium acetate), CaCl_2_, and labile Cd in pore water. These results were applied to predict the Cd bioavailability after the addition of colza cake to Cd-contaminated soil. Two commonly grown cash crops, wheat and maize, were selected for Cd accumulation studies, and were found to be sensitive to Cd bioavailability. Results showed that the addition of colza cake may inhibit the growth of wheat and maize. Furthermore, the addition of increasing colza cake doses led to decreasing shoot and root biomass accumulation. However, increasing colza cake doses did lead to the reduction of Cd accumulation in plant tissues, as indicated by the decreasing Cd concentrations in shoots and roots. The labile concentration of Cd obtained by DGT measurements and the traditional chemical extraction methods, showed the clear decrease of Cd with the addition of increasing colza cake doses. All indicators showed significant positive correlations (*p* < 0.01) with the accumulation of Cd in plant tissues, however, all of the methods could not reflect plant growth status. Additionally, the capability of Cd to change from solid phase to become available in a soil solution decreased with increasing colza cake doses. This was reflected by the decreases in the ratio (*R*) value of *C*_DGT_ to *C*_sol_. Our study suggests that the sharp decrease in *R* values could not only reflect the extremely low capability of labile Cd to be released from its solid phase, but may also be applied to evaluate the abnormal growth of the plants.

## 1. Introduction

Cadmium (Cd) is a non-essential element that is ubiquitously found in soil, water, and the atmosphere. Due to its high bioavailability and carcinogenic properties in humans, Cd has been blacklisted by the World Health Organization [1]. Cd is widely applied in industry for purposes such as electroplating, mining, and smelting, because of its metallic properties. This leads to the release of large amounts of Cd into soil through wastewater, irrigation, and sewage disposal [2]. The overuse of Cd-containing pesticides and fertilizers may also increase the concentration of Cd in the soil [3]. Due to its widespread occurrence, and its bioaccumulating and biomagnifying effect throughout tropic chains, Cd monitoring has become an area of increased research focus [4,5]. Excavation, phytoremediation, leaching, electro remediation, solidification, and stabilization are some of the many engineering and chemical techniques that have been proposed to reduce Cd bioavailability in contaminated soils [6]. However, all of these methods have apparent disadvantages. Due to its comparatively high destruction of native organisms in the soil environment, the excavation approach can easily disrupt ecosystem stability and therefore, is not an eco-friendly method [7]. Phytoremediation and phytoextraction are more eco-friendly-based approaches that may also be long-lasting and cost-effective. Using these approaches, Cd is accumulated in plant tissues and removed from the soil through the harvest of these plants [8,9]. However, due to the high selectivity of the biotic for the target elements, the capacity of plants to contain Cd, and the mobility of the element in soil, phytoremediation also does not supply an adequate solution to the reduction of Cd bioavailability in the soil [5]. Consequently, due to the severe implications of Cd contamination in the soil, the availability of a robust tool for the immobilization of Cd labile fractions is critical.

Previous investigations have shown performance inconsistencies of organic amendments in Cd contaminated soil. Some studies have revealed that the addition of organic materials might increase the ability of soil to bind the target element, making these contaminants available for removal through the remediation processes of sorption, precipitation, or complexation [9,10,11,12]. Yao *et al.* [5] have also indicated that typical organic pig manure could significantly reduce Cd toxicity interrestrial plants, such as wheat and maize. Additionally, the addition of organic materials could supply sufficient nutrients to plants and improve water retention in soil. Moreover, it was also reported that organic material can be beneficial by reducing phototoxicity and improving growth and survival of the biota [6,13]. Related studies have indicated that biochar, which may be the reactive group in the organic material, contains essential nutrients for plant germination and growth and, furthermore, could cover large areas to increase soil porosity and immobilize Cd fractions [14,15,16,17]. However, many investigations have also shown the increasing addition of the organic materials may enhance fraction mobility in soils and restrain biota growth [6,9,10]. Consequently, there are two prerequisites for organic materials that determine its applicability for soil amendment. One is that the organic amendment must be capable of reducing accumulated Cd in plant tissues during the plant’s lifetime. The other prerequisite is that the addition of the organic material must benefit the plant biomass. Accordingly, further investigations on the effectiveness of organic amendments for the remediation of Cd contaminated soil are necessary to give the overall understanding of organic amendment application. Colza cake is the collected residue of rapeseed after the refining process and is a common fertilizer for cultivation. However, there are no systematic investigations explaining its effect on metal mobility and bioavailability in Cd contaminated soils. Accordingly, colza cake was applied as the amendment material in this study.

Diffusive gradients in thin films (DGT), as an *in situ* measurement, has become an increasingly popular method for constructing eco-security and early-warning systems [18,19]. This technique, based on Fick’s first law, passively collects a target element while avoiding the influences of the base solution concentration and the surrounding environment [18]. The DGT device locally collects target element particles, while responding to fractions of resupplied labile species in a solution and the labile pools in the solid phase [19]. Consequently, the element concentration measured by the device is the mean concentration of the target element during deployment time. Due to the dynamic nature of the measurements, the DGT-measured concentration could effectively mimic the biota uptake process of metals such as Cu [20,21], Zn [20,22], Cd [21], and Hg [23,24]. Tian *et al.* [25] also demonstrated that the DGT obtained a concentration of a target element that was not influenced by the physiochemical properties of various metals and metalloids in soil, sediment, and water in the environment. Consequently, the use of DGT, as an *in situ* method, could dynamically reflect the bioavailability of Cd in soil. The traditional methods such as soil solution concentration [7], the free ion activity model, single or sequential extraction methods [26,27], and the isotope dilution exchange method are widely applied in the related investigations. These approaches have a lower time-cost ratio and simple operation procedures. These *ex situ* methods are of great significance when evaluating Cd bioavailability in a static manner [28,29]. Due to the different advantages of the *in situ* and *ex situ* methods, both the DGT technique and traditional chemical methods were applied in this study to reflect the immobilization of labile Cd fractions after the addition of organic material.

Typical cash crops, wheat and maize, were used to accumulate Cd. The DGT technique and five traditional static testing methods, including soil solution concentration and four commonly used chemical extraction methods (chelating extractant EDTA (ethylene diamine tetraacetic acid), acid extractant HOAc (aqua regia), and salt solutions CaCl_2_ and NaOAc (sodium acetate)), were selected to evaluate how these approaches reflect Cd bioavailability. The aim of this study was to systematically investigate Cd bioavailability in Cd-contaminated soil after the addition colza cake at varying concentrations. Pearson correlation coefficients, which determine differences between the bioavailable concentrations of Cd obtained by different indicators and the concentration of Cd accumulated in plant tissues, were used to compare bioaccumulation. The ratio of the DGT-measured concentration and the labile fractions in the soil solution under different addition concentrations of colza cake were also shown to reflect the resupply of labile Cd fractions. This further demonstrated the interaction between colza cake and labile Cd.

## 2. Experimental

### 2.1. Soil Samples and Incubation

Soil used in this study was collected from a suburb of Nanjing city in Jiangsu Province, China, which has adopted the “inter-planting mode” with paddy rice and wheat. Basic properties of this soil have been previously described [5]. Collected soil was typically yellow-brown in color, which is observed to be widespread in China. To ensure there was no point source of pollution, the collected soil samples were collected at a depth which was deeper than 20 cm. The soil samples were then sieved with a 2 mm stainless steel mesh after being air-dried at room temperature.

Soil subsamples were mixed with CdCl_2_ to achieve a 4.0 mg·kg^−1^ Cd concentration to create Cd-contaminated soil. Colza cake was then added as an organic amendment at eight different concentrations (g·kg^−1^) in the soil. The treatments were CK (control treatment without addition of Cd and colza cake), 0, 5.0, 10.0, 20.0, 40.0, 60.0, 80.0, and 100 g·kg^−1^. The CaCl_2_ solution and colza cake were thoroughly mixed with the sieved soil samples. To ensure the full aging of the experimental soil, all of the soils were incubated at room temperature for 36 months prior to the pot experiment. The soils were moistened with deionized water during the aging period, and were mixed once a week in order to ensure the full equilibration between naturally-occurring soil components and the amendment fractions.

### 2.2. Greenhouse Pot Experiment

Wheat and maize were grown in pots containing 750 g of the Cd-contaminated soils with different doses of colza cake. The soil moisture was maintained at 70% to 80% field water capacity with addition of deionized water. Fifteen wheat seeds and seven maize seed were sown in every experimental soil type with three replicates for every concentration. After germination, plant numbers were reduced to 10 wheat seedlings and five maize seedlings in every pot. All plants were grown under the natural day-night cycle in a greenhouse with no additional nutrients. After six weeks, all plants were harvested and separated into shoots and roots. Plant shoots were cut with scissors, cleaned with tap water, and further rinsed with deionized water to remove soil. Harvested plant roots were placed in a 20 mmol·L^−1^ EDTA solution for 15 min and then washed with deionized water in order to remove fine particles adsorbed into the root surface. Shoots and roots were dried in an oven at 70 °C for 4 h to destroy chlorophyll and then the temperature was reduced to 50 °C until the tissue weight was constant. Cadmium concentrations in the plant tissues were determined by atomic absorption spectrophotometry (Z-81001, Hitachi Ltd., Hitachi, Japan) following digestion with HNO_3_/HClO_4_. To ensure accuracy, increase precision, and reduce experimental error (<5%), quality control (GBW(E) 081581, Bureau of Standard Measurement, Beijing , China) of the analytical method was conducted every ten samples using a certified standard solution [5]. After harvest, the remaining soil samples were air-dried at room temperature and then sieved with a 2 mm stainless steel mesh for analysis of various parameters (described below).

### 2.3. Analytical Methods of the Bioavailable Cd in Soils

#### 2.3.1. *In Situ* DGT Measurement

The principle of DGT is defined by the diffusion of dissolved species through a well-defined gel and subsequent accumulation on an ion-exchange resin. To control and astrict the accumulation concentration of the target element, a diffusion gel and a filter membrane are commonly used as the diffusive layer. The resin gel, which serves as a binding agent, is incorporated into a second polyacrylamide gel. The concentrations of the target elements are measured, including the fractions in pore water near the device surface and the fractions resupplied from solution, as well as that continuously released from the sediments.

**Figure 1:**
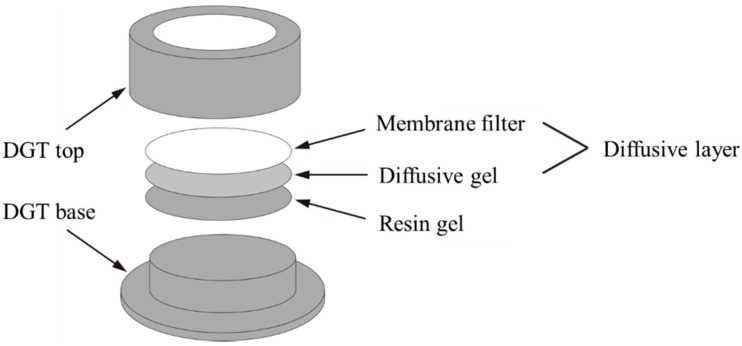
Component of a DGT device.

A standard piston-type DGT device purchased from DGT Research Limited Corporation [30] was composed of a plastic base, a resin gel, a diffusive gel, a protective membrane filter, and a plastic cap (Figure 1). The diffusive gel (0.8 mm thick) was prepared with 15% acrylamide and 0.3% agarose-derived cross-linker following a published procedure [18]. The resin gel (0.4 mm thick) was synthesized by infusing Chelex-100 into the diffusive gel. In the DGT assembly, the resin gel was sequentially covered by a diffusion gel and a 0.13 mm cellulose nitrate filter membrane (0.45 μm pore size. Whatman, Maidstone, UK) [18].

DGT device application in soil was based on a published procedure by Luo *et al.* [7]. The procedure was divided into the following five steps:

1. Subsample Moisture Adjustment

To ensure there was a complete equilibrium between fractions and soil, an 80 g subsample of each soil sample was placed in a plastic pot and maintained to 80% maximum water holding capacity (MWHC) with deionized water at 25 °C for 48 h. Water content was replenished to 80% MWHC for 24 h before DGT deployment.

2. DGT Accumulation

**Figure 2:**
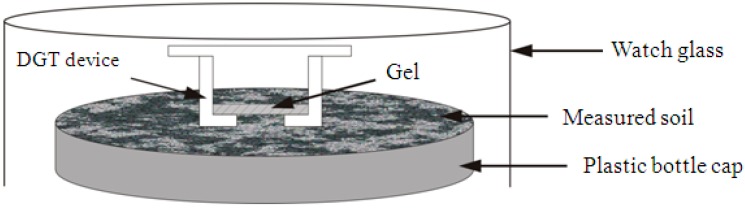
Schematic view of DGT deployment in soil.

The DGT devices were gently pressed on to the surface of each subsample, in the plastic pot, and remained in position for 24 h. To ensure that moisture was stable in the subsample, a cultivating dish was used to cover the DGT device (Figure 2). Temperature was controlled to be constant at 25 ± 1 °C during DGT deployment.

3. Retrieval of the DGT Device

All DGT devices were retrieved after 24 h. Deionized water was applied to wash the filter membrane in order to remove soil particles that had adhered to the DGT device surface. Rinsed DGT devices were sealed in a Ziploc bag in order to avoid drying of resin within the device.

4. DGT Elution

After the DGT device was disassembled, the resin gels were transferred into a centrifuge tube with 1 mL of 1 mol·L^−1^ nitric acid and then shaken for 24 h. Cd concentrations eluted by nitric acid were measured using flame atomic absorption spectrophotometry (Hitachi Z-81001).

5. Calculation of the DGT-Measured Concentrations

The DGT-measured mass of Cd (M) in the resin gel was calculated according to the Equation (1) when a known volume of eluting solution was used for elution (*V_s_*):

(1) $$M=\frac{C_{e}\left(V_{g}+V_{S}\right)}{f_{e}}$$

where *V_g_* is the volume of the resin gel, 0.2 mL, and *f_e_* is the elution factor, 0.8 [18].

The concentrations of Cd measured by DGT (*C_DGT_*) were calculated using Equation (2):

(2) $$C_{DGT}=\frac{M\Delta g}{DAt}$$

where *M* is the accumulated mass of Cd over the deployment time (ng). Δ*g* is the thickness of the diffusive layer (cm). D is the diffusion coefficient of Cd in the diffusive layer (cm^2^·s^−1^), which has been reported in a DGT website (www.dgtresearch.com). A is the area of DGT exposure window (cm^2^) and t is the time of DGT deployment (s) [18].

#### 2.3.2. *Ex Situ* Measurement

##### Single Extraction Methods

Four widely-sed single extraction methods were selected to extract the labile Cd fractions in Cd-contaminated soil after the addition of varying concentrations of colza cake. The chemical agents used during extractions were 0.05 mol·L^−1^ EDTA, 0.11 mol·L^−1^ HOAc, 1 mol·L^−1^ NaOAc, and 0.01 mol·L^−1^ CaCl_2_. Among these four extraction agents, the common chelating agent EDTA had the strongest binding strength with complex ion fractions existing in the soil. The use of 0.11 mol·L^−1^ HOAc was reported to influence biota growth and it was the first step in a three-step extraction procedure recommended by the Standards, Measurements, and Testing Program (SM and T) of the European Commission [31]. NaOAc and CaCl_2_ are typically used in ion exchange-based methods because, as weak substitution agents, they could be used to evaluate labile fractions through the replacement of their cation.

To avoid extractant overheating, all solutions were centrifuged at 3000 *g* for 20 min at 25 °C. The supernatants were then filtered with plastic strainers, transferred to 10 mL centrifuge tubes with HNO_3_, and subsequently stored at 4 °C prior to analysis. All extraction procedures were conducted in triplicate. Details of these operations are listed in Table 1.

##### Soil Solution Concentration

Soil subsamples from which the deployed DGT were collected were centrifuged at 10,000 g for 20 min at 25 °C to obtain a soil paste. A 0.45 μm pore size cellulose nitrate filter membrane was used to filter the paste and ensure the purity of the samples. The solutions were acidified using HNO_3_.

The concentration of Cd in extracts and soil solutions was measured using atomic absorption spectrophotometry (Hitachi Z-81001). Certified standard solutions and duplicates of all samples were applied to ensure accuracy and precision. For each test of the standard solutions, all obtained metal concentrations agreed with the certified values within the expected range of experimental error (<5%). Relative standard deviations based on the mean values obtained for each sample type were less than 5% [5].

### 2.4. Data Analyses

Statistical analyses were performed using the SPSS statistical package (version 10.0 for Windows, IBM, New York, NY, USA). The data for shoot and root dry weights were tested at a significance level of *p* < 0.05 by one-way ANOVA analysis of variance. The relationship between various bioavailable indicators of Cd, measured by six methods in soils, and plant Cd uptake were investigated using the Pearson correlation coefficient.

## 3. Results and Discussion

### 3.1. Plant Growth and Accumulation in Response to Colza Cake Exposure

**Figure 3:**
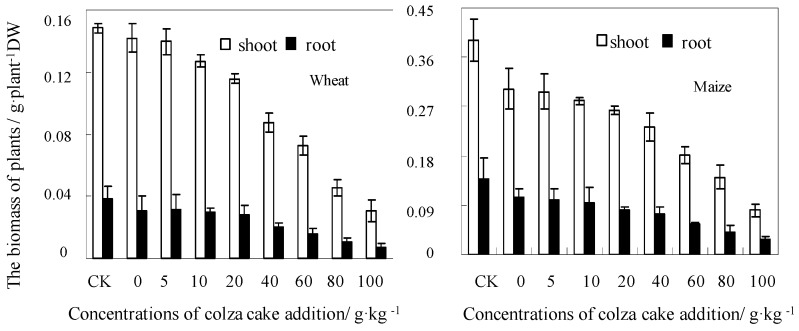
Effects of colza cake addition on the biomass (expressed as dry weight) of wheat and maize in Cd-contaminated soils.

**Figure 4:**
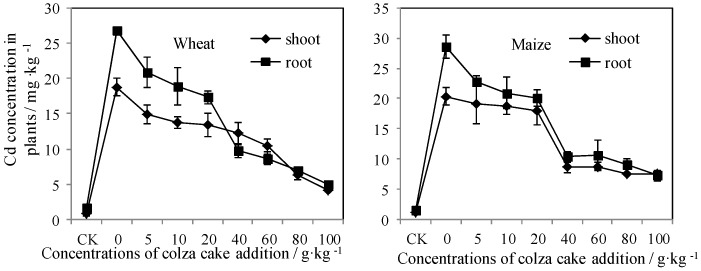
Cadmium concentrations in plant tissues of wheat and maize after the addition of colza cake in Cd-contaminated soils.

Colza cake is a commonly applied organic material for fertilization in China. It is rich in N and P, and has sufficient protein content, which is comprised of approximately 30% crude protein and 25% easy to decompose protein [35,36]. As shown in Figure 3, the growth of wheat was not affected by single Cd treatment (4.0 mg·kg^−1^), however, the growth of maize was clearly inhibited by single Cd addition, its biomass of shoot and root were significantly (*p* < 0.05) decreased by 21% and 19%, respectively, compared with the control plant tissues (CK). Moreover, colza cake addition in Cd-contaminated soils may inhibit the biota growth to a large extent, which was indicated by the decreased biomass of wheat and maize. Similar investigations have indicated contradictory conclusions, where the addition of organic materials, such as straw manure, which could significantly promote the growth of radish in Cd and Cu contaminated soil, and such as chicken manure amendments, which could increase Pb/Cu accumulation in *Brassica chinensis* and sunflower [37]. In addition, these results provide contrary evidence to the varying trend of Cd accumulation in plant tissues. The concentration of Cd in the tissues of wheat and maize are displayed in Figure 4. Colza cake, as an additive, clearly restricted Cd accumulation in the shoots and roots of both plants and its inhibitory effects increased with increasing doses. Cd concentrations in shoots and roots were decreased by 71% and 83% for wheat, and by 61% and 77% for maize, respectively, when the added amount of colza cake was at least 100 g·kg^−1^. Previous investigations have indicated the addition of organic materials to soil may theoretically increase plant biomass, could optimize physicochemical properties, such as the nutrient concentrations of Fe, Mn, Cu, and Zn, as well as soil acidity and alkalinity, which benefit biota growth [11,38]. However, due to the characteristics of heat production, the application of colza cake could easily cause the loss of elements, such as N, P, and K. In addition, the extremely high concentration of colza cake could also lead to withering and death of roots. As a result, the biomass of wheat and maize plants were sharply decreased after the addition of high concentrations of colza cake (60–100 g·kg^−1^) [11]. In view of this phenomenon, we thought that the effects of organic amendments to the Cd contaminated soil could be dependent upon the release mechanism of the fertilizer. Similar studies have shown that colza cake may release superabundant nutrients to become dissolved into the soil solution, which led to changes in the concentration gradients between the water and cells. This causes extreme water loss within cells and results in plasmolysis [13,14].

### 3.2. Bioavailable Cd Reflected by a Dynamic Measurement

In order to provide a robust tool to evaluate the effects of organic amendments on Cd concentrations in contaminated soil, a novel *in situ* technique using DGT was applied to mimic the accumulation of Cd in plant tissues. DGT is a rapid and dynamic technique based on the Fick’s first law, which is used to measure concentration of a target element. This depends upon diffusion-controlled fluxes of a solute from the surface of the DGT device to the resin gel (binding phase). The DGT technique measures labile species, with the exclusion of kinetically-inert organic species, large colloids, and strong organic-metal complexes. The dynamic nature of its measurements ensures that information obtained from contaminated soils is a result of the average concentration during the DGT device deployment [18,39]. These characteristics also aid in the avoidance of analytical errors, which can arise due to the uneven distribution of labile fractions [7]. Therefore, the labile concentration measured by DGT could serve as a model for the ability of organisms to uptake the target element from the extract solution supplied by the solid state of the element in soil, although DGT obtained concentration information was limited and controlled by the diffusion quantification in the diffusion layer [40]. Several studies have indicated that the DGT method has great advantages in predicting the bioavailability of metals, such as Zn, Pb, Cu, and As, after the addition of organic materials, and was sensitive to changes in bioavailable fractions without the influence of the complex physic-chemical properties [20,24,41]. Consequently, DGT can be used as a robust tool to evaluate metal bioavailability in soils and evaluate metal accumulation by plants after the addition of colza cake.

**Figure 5:**
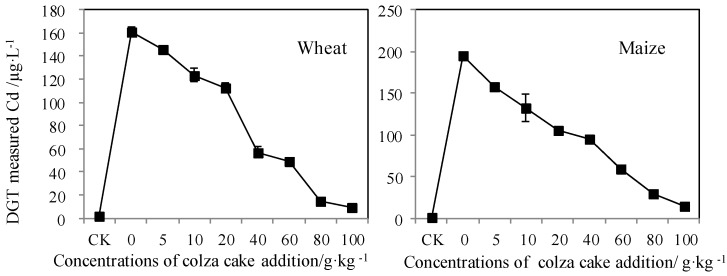
DGT-measured concentrations (*C*_DGT_) of Cd after the addition of colza cake in Cd-contaminated soils grown by wheat and maize, respectively.

As presented in Figure 5, the DGT-labile concentrations of Cd (*C*_DGT_) in soils, in which wheat and maize were grown, significantly decreased with the addition of increasing doses of colza cake. This trend positively correlated with lower Cd accumulation in shoots and roots of biota after addition of colza cake. Results of DGT indicated that the addition of colza cake can be effective in reducing the concentration of bioavailable Cd in Cd-contaminated soils. The increased proportion of organic-bound Cd in soil resulted in a significant decrease in Cd mobility. Consequently, it was concluded that the application of the DGT method could reflect the bioavailability of Cd in a soil solution after the addition of colza cake, and/or the decrease in the rate of resupply of Cd to solution from the solid phase. However, the excessive addition of colza cake could lead to growth retardation in wheat and maize and, as a result, the DGT method could not predict plant growth status. Many investigations have indicated that the simulation of DGT has neglected the microenvironment in the vicinity of the rhizosphere, and the effects of naturally-occurring microorganisms on roots [40].

### 3.3. Bioavailable Cd Reflected by Static Measurements

#### 3.3.1. Soil Solution Concentration of Cd

**Figure 6:**
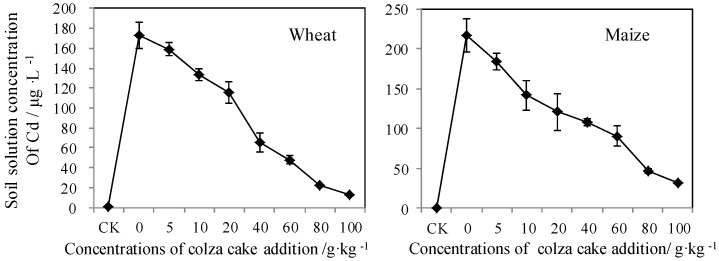
Soil solution concentrations (*C*_sol_) of Cd after the addition of colza cake in Cd-contaminated soils grown by wheat and maize, respectively.

Due to the direct uptake of mineral elements by the roots of biota from the soil solution, several researchers have indicated that measuring total dissolved Cd in soil solutions is a more direct method to evaluate the potential for Cd accumulation in biota [42,43,44]. Until now, heavy metals that accumulate in biota were divided into two categories; heavy metal fractions that are metabolically-available, and heavy metal fractions that are no longer bioavailable [45]. Accordingly, dissolved Cd may be represented by the accumulated Cd concentration in biota, but it could not represent the relative toxicity to the biota. Moreover, there were few reports on the effect of soil solution which was applied to evaluate the Cd bioavailability after the addition of organic materials. Further investigation was necessary to establish a comprehensive understanding of soil solution evaluation. As shown in Figure 6, the Cd concentration in a soil solution (*C*_sol_) after planting two plants considerably decreased with the addition of increasing doses of colza cake. This trend was similar to the trend seen using DGT. The addition of colza cake could significantly decrease the dissolved concentration of Cd when compared to Cd-contaminated soils with no colza cake addition. The concentration of Cd was reduced by 11% to 87% for wheat, and by 8% to 91% for maize, when the added amounts varied from 5.0 to 100 g·kg^−1^. Beesley *et al.* [11] produced similar results when they reported a significant decrease in Cd and Zn concentrations in pore water with the addition of biochar and green waste compost. As indicated in this study, the Cd concentration in soil solutions could not reflect the growth status of wheat and maize after the addition of colza cake. Colza cake, without composting, was difficult to dissolve in a soil solution. As a result, excess colza cake may inhibit the clearing of the soil particles, which may lead to the build-up of an anaerobic environment near roots, the lack of nutrient availability, and overall biota growth retardation.

#### 3.3.2. Bioavailable Cd Reflected by Traditional Extraction Measurements

**Figure 7:**
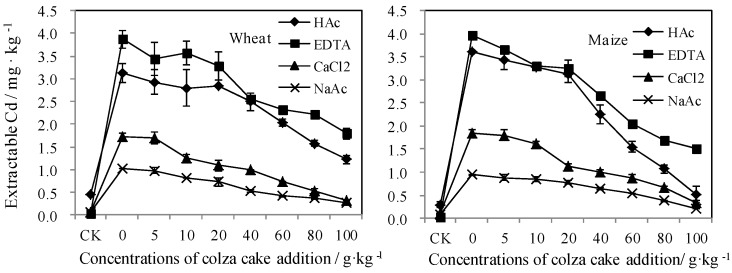
The bioavailable concentrations of Cd measured by different extraction methods after the addition of colza cake in Cd-contaminated soils grown by wheat and maize, respectively.

The chemical agents used in traditional extraction methods could be used to evaluate the mobilization of different Cd fractions in Cd-contaminated soil after the addition of organic amendments [8,9,46]. However, many investigations have reported that measurements of heavy metal concentrations using these chemical agents were arbitrary and had no direct connection with fraction accumulation in biota [26,47]. To further verify the utilization of these extraction measurements, as well as to investigate the variations in Cd concentrations with the addition of colza cake, it was essential to evaluate the effects of chemical agents on Cd bioavailability. The concentrations of Cd measured by the four extraction chemicals (HOAc, EDTA, NaOAc, and CaCl_2_) are shown in Figure 7. The Cd concentration, as measured using the four extraction chemicals, decreased with the addition of increased doses of colza cake. Based on the varying intensity of the extractions, the four reagents were applied to selectively release fractions on exchange sites or carbonate phase, and release metals bound to soil organic matter [29,41,47,48,49]. For the soils in which wheat and maize were grown, variation in the extracted amounts of Cd with four extractants had similar trends as results for Cd concentrations in soils measured by DGT, as indicated by the gradual decreases in Cd concentration with increasing additions of colza cake. However, because of the higher percentage of fraction redistribution and readsorption, the decreases in Cd concentration after colza cake addition seen with the use of NaOAc and CaCl_2_ occurred at a slower pace in comparison to EDTA and HOAc.

Among the four extractants, EDTA extracted the largest amount of Cd from soils in comparison to HOAc, NaOAc, and CaCl_2_. Since EDTA was a chelating agent, it was strong enough to extract Cd, which is bound to organic materials in the oxidation state generated by secondary clay minerals [26]. Following EDTA, it was hypothesized that HAc would extract more Cd from soils than the remaining two agents. HOAc acts as an acid extractant that can partially extract organic matter bound metals and release most bioavailable fractions that are coupled with calcium carbonate and minerals [50,51]. NaOAc and CaCl_2_ are weak neutral salts that can extract metal components on mineral surfaces based on the cation exchange principle [26,27]. Due to the low impact on the plant growing environment, the bioavailable fractions extracted by these solutions have been suggested to represent active sources in soils for plant uptake [42].

In this study, the decreasing Cd concentrations in EDTA, HOAc, NaOAc, and CaCl_2_ extracted solutions, as well as in soil solutions, indicated that the bioavailable species of Cd may be easily translated to inert form after the addition of colza cake. This was in agreement with the decreases observed using the DGT method (Figure 5). Coupled with dynamic measurements made using DGT, these static data, including soil solution Cd concentration measurements determined using the four extraction agents, further implied that the bioavailable fractions in soils were reduced after mixing colza cake with soil [52].

### 3.4. The Inhibition to Cd Bioavailability Caused by Colza Cake

As shown in Table 2, the linear relationships between Cd concentrations in plants and bioavailable concentrations of Cd were analyzed using the Pearson Correlation Coefficient (two-tailed). All these methods used to measure Cd concentrations in soil showed significantly positive relationships (*p* < 0.01) with accumulated Cd in plant tissues. Comparing the correlation coefficients obtained by the DGT technique with those obtained by traditional approaches revealed no substantial differences. Several investigations have revealed the discernible advantages of using the DGT device for evaluating heavy metal bioavailability in soil compared to the traditional extraction methods [18,20,49]. The indiscrimination between the results of DGT-measured Cd concentrations and Cd concentrations obtained from the traditional methods were due to the influence of colza cake.

The mobilization of target elements in the soil dramatically impacted their bioavailability, which was indicated by the diffusion intensity of the resupplied element from the solid phase to soil solution [20,50,51]. Accordingly, the ratio of *C*_DGT_ to *C*_sol_ (*R*) was used to reflect the extent to which metals were resupplied to the soil solution from solid phase [19]. The *R* value varies from 0 to 1, with a value close to 1 indicating a very rapid resupply of the element (*i.e.*, the sustained case) and a value close to 0.1 indicating very slow resupply (the diffusive case). A medium *R* value between 1 and 0.1 is referred to as the partial case [51]. As listed in Table 3, the *R* values gradually decreased with increasing additions of colza cake. However, the obvious decreases in this trend were observed when the additions of colza cake were up to 60 g·kg^−1^. Yao *et al.* [5] used pig manure to mitigate the bioavailability of Cd in the same Cd-contaminated soil. The R values in their study also showed a decreasing trend with the addition of pig manure, but the *R* values decreased at a significantly slower pace than the *R* values observed after the addition of colza cake. We speculated that the watertight nature of colza cake may be responsible for the extremely small *R* values when the addition concentrations of colza cake were more than 60 g·kg^−1^.

## 4. Conclusions

Although the addition of colza cake could clearly reduce Cd bioavailability, the biomass of wheat and maize were significantly decreased with the addition of colza cake at increasing doses. Consequently, colza cake amendments may not be an effective method for the remediation of Cd-contaminated soil. The bioavailable Cd obtained by DGT and traditional chemical methods both showed similar variations with the accumulated Cd in plant tissues, as indicated by the positive significance analysis (*p* < 0.01). However, all of the selected indicators in this study could not reflect the growth status of the biota. These indicators gave a one-sided evaluation based on Cd bioavailability, while ignoring the negative effects of colza cake on the biota. Although that *R* values (the ratio of *C*_DGT_ to *C*_sol_) could not only evaluate Cd bioavailability after the addition of colza cake but also reflect the growth condition of the wheat and maize in this study. However, due to the rate of the adsorption and desorption of the sorbed Cd fractions, future research will be necessary for the performance of the *R* value.

## Figures and Tables

**Table 1:** The procedures of four extraction methods adopted in this study.

Extractants	Procedure	References
EDTA	2.0 g of soil was extracted with 20 mL of 0.05 mol·L^−1^ EDT Aadjusted using an ammonia solution to pH = 7.0 and shaken for 2 h	Wear and Evans (1968) [32]
HOAc	0.5 g of soil was extracted with 20 mL of 0.11 mol·L^−1^ HOAc and shaken for 16 h (overnight)	Houba *et al.* (1996) [29]
NaOAc	4.0 g of soil was extracted with 20 mL of 1 mol·L^−1^ NaOAc and shaken for 2 h	Kaplan *et al.* (2009) [33]
CaCl_2_	2.0 g of soil was extracted with 20 mL of 0.01 mol·L^−1^ CaCl_2_ and shaken for 3 h	Novozamsky *et al.* (1993) [34]

**Table 2:** The Pearson correlation coefficients (*r*) of the 24 samplers between Cd concentrations in the plant tissues and bioavailable concentrations of Cd measured by six methods.

Plant Species	Plant Tissues	DGT	Soil Solution	HAc	EDTA	NaAc	CaCl_2_
wheat	shoot	0.971 **	0.967 **	0.883 **	0.966 **	0.956 **	0.890 **
	root	0.979 **	0.979 **	0.894 **	0.974 **	0.975 **	0.934 **
maize	shoot	0.974 **	0.972 **	0.954 **	0.961 **	0.971 **	0.933 **
	root	0.970 **	0.962 **	0.944 **	0.969 **	0.936 **	0.949 **

** Correlation is significant at the level of *p* < 0.01.

**Table 3:** The calculated ratio (*R*) values of the DGT-measured concentrations (*C*_DGT_) of Cd to soil solution concentrations (*C*_sol_) of Cd with increasing addition of colza cake in soils.

Colza Cake Levels in Soil (g·kg^−1^)	Wheat	Maize
CK	0.83	0.85
0	0.73	0.74
5.0	0.71	0.69
10.0	0.67	0.68
20.0	0.63	0.65
40.0	0.51	0.52
60.0	0.37	0.29
80.0	0.31	0.21
100.0	0.27	0.16

CK represents the control group, *R* = *C*_DGT_/*C*_sol_.
